# Evaluation of enterotoxigenic *Bacteroides fragilis* correlation with the expression of cellular signaling pathway genes in Iranian patients with colorectal cancer

**DOI:** 10.1186/s13027-023-00523-w

**Published:** 2023-08-29

**Authors:** Leila Dadgar-Zankbar, Aref Shariati, Narjess Bostanghadiri, Zahra Elahi, Shiva Mirkalantari, Shabnam Razavi, Fatemeh Kamali, Davood Darban-Sarokhalil

**Affiliations:** 1 Department of Microbiology, School of Medicine, Iran University of Medical Sciences, Tehran, Iran; 2 Molecular and Medicine Research Center, Khomein University of Medical Sciences, Khomein, Iran; 3 Student Research Committee, Khomein University of Medical Sciences, Khomein, Iran; 4 Iran National Tumor Bank, Cancer Institute of Iran, Tehran University of Medical Sciences, Tehran, Iran

**Keywords:** *Bacteroides fragilis*, Colorectal cancer, WNT/β-catenin, TP53, BCL2

## Abstract

**Background:**

Colorectal cancer (CRC) is one of the most common cancers all over the world, and dysbiosis in the gut microbiota may play a role in colorectal carcinogenesis. *Bacteroides fragilis* can lead to tumorigenesis by changing signaling pathways, including the WNT/β-catenin pathway. Therefore, in the present study, we investigated the correlation between the enterotoxigenic *B. fragilis* amount and the expression of signaling pathway genes involved in CRC.

**Materials and methods:**

*B. fragilis* was determined in 30 tumors and adjacent healthy tissues by the qPCR method. Next, the relationship between enterotoxigenic *B. fragilis* and the expression of signaling pathway genes, including CCND1, TP53, BCL2, BAX, WNT, TCF, AXIN, APC, and CTNNB1 was investigated. Additionally, possible correlations between clinicopathological features of the tumor samples and the abundance of *B. fragilis* were analyzed.

**Results:**

The results showed that *B. fragilis* was detected in 100% of tumor samples and 86% of healthy tissues. Additionally, enterotoxigenic *B. fragilis* colonized 47% of all samples, and *bft-1* toxin was the most frequently found isotype among the samples. The analysis showed that the high level of *B. fragilis* has a significant relationship with the high expression of AXIN, CTNNB1, and BCL2 genes. On the other hand, our results did not show any possible correlation between this bacterium and the clinicopathological features of the tumor sample.

**Conclusion:**

*B. fragilis* had a higher abundance in the tumor samples than in healthy tissues, and this bacterium may lead to CRC by making changes in cellular signaling pathways and genes. Therefore, to better understand the physiological effects of *B. fragilis* on the inflammatory response and CRC, future research should focus on dissecting the molecular mechanisms by which this bacterium regulates cellular signaling pathways.

## Introduction

Colorectal cancer (CRC) is the third most common lethal cancer worldwide and the fourth most commonly diagnosed in both sexes [1, 2]. By 2030, the global incidence of CRC is expected to rise to 2.2 million new cases and 1.1 million deaths [3]. This cancer had been associated with people over 50, but recently its rate has increased in people between the ages of 40 and 49 [1, 4].

The etiology of CRC is complex and multifactorial; however, genetic and environmental factors play an important role in CRC [5]. Environmental factors include western diet habitats, alcohol drinks, red and processed meat consumption, smoking, being overweight, and a lack of physical activity [6, 7]. Other risk factors for CRC are gastrointestinal diseases, such as inflammatory bowel disease (IBD), which cause inflammation, increased production of reactive oxygen species (ROS), and damage to the intestinal mucosa. Noteworthy, recently published studies reported that gut microbiota also plays an important role in the carcinogenesis of CRC [5, 8].

The gut microbiota is essential for intestinal homeostasis and health by participating in nutrition, metabolism, and protection. They also have anti-tumor, anti-inflammatory, and anti-bacterial actions, producing chemicals such as vitamins, niacin, and amino acids [9, 10]. A healthy microbiome has a high species diversity and is mainly composed of *Firmicutes*, *Bacteroidetes*, and *Actinobacteria* [11, 12]. When the composition of the intestinal microbiota is altered, dysbiosis occurs and could increase the risk of CRC in humans [13]. The results of studies have shown that pathogenic bacteria such as *Fusobacterium nucleatum, Escherichia coli*, and *Bacteroides fragilis* are predominant in CRC patients’ intestines [4, 12]. *B. fragilis* is an anaerobic bacterium that has been identified in the intestines of 80% of children and adults and comprises less than 1% of the total intestinal microbiome. There are two enterotoxigenic and non-enterotoxigenic types of *B. fragilis*, and according to studies, the first type plays a role in gastrointestinal diseases, including CRC [14].

In addition to dysbiosis, toxins produced by some bacteria can play an important role in the progression of cancer. In fact, bacteria use these toxins to make the host cell’s environment favorable [15]. To this end, enterotoxigenic *B. fragilis* (ETBF) produces a 20 KD zinc-dependent metalloprotease called *B*. *fragilis* toxin (*bft*) with three isotypes, including *bft-1*, *bft-2*, and *bft-3* [16, 17]. Recent studies reported that long-term colonization of ETBF in clonal epithelial cells increases the risk of CRC [18, 19]. It has been shown that *B. fragilis* toxin exerts its tumorigenic effect by cleaving the extracellular domain of E-cadherin. The toxin binds receptors on epithelial cells then this cause the transfer of the signals into cell and the extracelluar domain of E-cadherin cleaves [20]. E-cadherin is a tumor suppressor protein that maintains the integrity of epithelial cells and can bind to β-catenin through its intracellular domain [15, 20, 21]. The cleavage of extracellular domain of E-cadherin causes the accumulation of β-catenin in the cytosol [18, 21]. Under normal conditions inside the cell, β-catenin is degraded by tumor suppressor proteins complex, including adenomatous polyposis coli (APC), glycogen synthase kinase-3 (GSK-3), and axis inhibitor (AXIN). But in the presence of toxin, free β-catenin in the cytosol accumulates then translocates to the nucleus, binds to the transcription factor/lymphoid enhancer binding factor (TCF/LEF), and increases the transcription of the proto-oncogene cellular c-MYC (MYC) and CCND1 genes that encode cyclin D1 protein [20, 21].

Additionally, *bft* could lead to tumorigenesis through the activation of pro-inflammatory cytokines such as interleukin-8 (IL-8) and up-regulation of signaling pathways such as mitogen-activated protein kinases (MAPKs) and Wnt family member (WNT) [15, 17, 18, 22]. The tumor protein P53 (TP53) gene is one of the genes that encode the tumor suppressor protein p53, which is one of the cell cycle checkpoint proteins. If there is a mutation or change in it, it increases proliferation and tumorigenesis [23]. Therefore, as mentioned, *B. fragilis* could cause tumorigenesis through interference in different cellular signaling pathways. To this end, in the present study, we evaluated the relationship between *B. fragilis* and its different toxin isotypes and the cellular signaling pathways of CRC in Iranian patients.

## Materials and methods

### Sample preparation

In this study, 30 colorectal carcinomas and adjacent healthy tissue were provided by the Iran National Tumor Bank, which is founded by the Cancer Institute of Tehran University of Medical Sciences, for Cancer Research between February 2019 and January 2021. Patients were not considered who met the following criteria: (a) had a tumor type other than adenocarcinoma in the colon; (b) had received probiotics, antibiotics, chemotherapy, or radiation therapy before surgery; and (c) had concomitant malignancies in other organs. All specimens were obtained after resection of the primary tumor or before the initiation of treatment. Following surgical removal of the tissues, the samples were transported from the operating suite to the pathology unit. There, they were evaluated by the pathologist, who was blind to the clinical and molecular information. RNAlater Reagent (QIAGEN) was used to fix a portion of the control mucosa samples as well as a portion of one of the tumorous tissues, and they were then frozen and maintained at -70° C before nucleic acids extraction. From patients’ records and case report forms, all clinical data and essential information such as gender, age, and histopathological characteristics were collected. Noteworthy, the Iran University of Medical Sciences Ethics Committee gave its approval to the study’s protocol. Informed consent was obtained from all patients.

### DNA extraction

The DNA of the healthy and tumor tissues (25 mg of each tissue) was extracted by the FavorPrep DNA Mini Kit (Favorgen). Following extraction, DNA quality, and quantity were determined using an OD (260) spectrophotometer and an agarose gel. The validated DNA extracts were then stored at -20° C for further analysis and qPCR.

### RNA extraction and cDNA synthesis

Total RNA was extracted from CRC and normal tissues using a FavorPrep RNA purification mini kit (Favorgen, Ping Tung, Taiwan). Following extraction, RNA quantity and quality were determined using an OD (260) spectrophotometer and an agarose gel. cDNA synthesis was done using the cDNA synthesis kit (Yekta Tajhiz Azma, Tehran, Iran). Then, the synthesized cDNA was stored at -20° C for further analysis and qPCR.

### Quantitative PCR for *B. fragilis*

TaqMan primer-probe sets were used to identify the *B. fragilis* 16 S rDNA gene sequence (Table 1). NCBI BLAST databases were used to assess the specificities of the primers and probes. Each reaction mixture contained 0.5 µM of the probe, 1 µM of each primer, 5.5 µl of Universal Probe Ex Taq PCR Master Mix (Ampliqon, Denmark), and 3 µl of extracted DNA, for a total volume of 20 µl. qPCR was carried out by the Rotor-Gene 6000 real-time PCR cycler (Qiagen Corbett, Hilden, Germany) using the following program: an initial holding at 95° C for 15 min, followed by 40 cycles of denaturation at 95° C for 15 s, and annealing/extension at 59° C for 30 s. The reaction mixture components that did not include genomic DNA were used as a negative control in all tests. Noteworthy, all of the assays were performed in triplicate in a single patch, and the results were averaged; hence, the data that are provided in this paper are the mean values of the qPCR analyses that were performed in triplicate.

Solute carrier organic anion transporter family member SLCO2A1 was used as an internal control, and by using the 2 ^−ΔΔCT^ method (where CT is the difference between the average CT value of *B. fragilis* and the reference gene), the amount of *B. fragilis* in each sample was determined as a relative unitless value and then normalized to SLCO2A1. This was done under the methodology that was described earlier [24, 25].

### Signaling pathway gene expression

In the present study, we used qPCR to evaluate the expression of WNT, CTNNB1, AXIN, TCF, APC, TP53, B-cell leukemia/lymphoma 2 (BCL2*)*, BCL2-associated X protein (BAX*)*, and CCND1 genes. All of the primers that were used in this study are presented in Table 1. The levels of expression of the mentioned genes were evaluated in triplicate reactions using qPCR and melt curve analysis. qPCR was carried out by the SYBR-Green master mix and the Rotor-Gene 6000 real-time PCR cycler. The reaction mixture contained: 0.5 µM of each primer, 5.25 µl SYBR-Green master mix, 3 µl of synthesized cDNA, and 3.25 µl H_2_O. The following protocol was applied: an initial holding at 95° C for 15 min, followed by 40 cycles of denaturation at 95° C for 15 s, annealing for 30 s at different temperatures for each gene, and extension at 72° C for 25 s. The SLCO2A1 gene was used for internal control, and mRNA levels were quantified using the 2 ^−ΔΔCT^ approach (Table 1) [26].

### Detection of *B. fragilis* enterotoxin isotypes

The enterotoxin isotype-encoding genes (*bft-1*, *bft-2*, and *bft-3*) were detected in *B. fragilis*-positive samples by PCR (Table 1). Each reaction mixture contained: 1 µM of each primer, 3 µl of extracted DNA, 12.5 µl 2x red PCR master mix (Amplicon, Denmark), and 7.5 µl H_2_O. The protocol was applied by peqStar (Peqlab, Germany): initial denaturation at 94° C for 5 min, followed by 36 cycles of denaturation at 94° C for 45 s, annealing at 52° C for *bft-1*, 50.5° C for *bft-2*, and 53.5° C for *bft-3* for 30 s, extension at 72° C for 45 s, and final extension at 72° C for 5 min [18].

**Table 1:** Specific primers and TaqMan probes were utilized in the present research

Target gene	Primer/Probe	Oligonucleotide sequence (50e30)	Product size (bp)	Ref
*B. fragilis*	Primer R Primer F Probe	CGGAATCATTATGCTATCGGGTA CGAGGGGCATCAGGAAGAA CTTGCTTTCTTTGCTGGCGACCG	136	[24]
*bft-1*	Primer R Primer F	GAACCTAAAACGGTATATGT CCT CTT TGG CGT CGC	190	[27]
*bft-2*	Primer R Primer F	GAACCTAAAACGGTATATGT CGC TCG GGC AAC TAT	175	[27]
*bft-3*	Primer R Primer F	GAACCTAAAACGGTATATGT TGT CCC AAG TTC CCC AG	287	[27]
CCND1	Primer R Primer F	CCTCCTTCTGCACACATTTGAA GCTGCGAAGTGGAAACCATC	135	[28]
TP53	Primer R Primer F	TCATCCAAATACTCCACACGC CAGCACATGACGGAGGTTGT	125	[28]
BCL2	Primer R Primer F	CAGAGACAGCCAGGAGAAATCA TCGCCCTGTGGATGACTGA	134	[29]
BAX	Primer R Primer F	TGCCACTCGGAAAAAGACCTC TTTTGCTTCAGGGTTTCATCCA	155	[30]
WNT	Primer R Primer F	GTGGTCCAGGATAGTCGTGC GCGTGTTAGTGTCCAGGGAG	110	[31]
TCF	Primer R Primer F	GTTCATGTGGATGCAGGCTAC GGCTATGCAGGAATGTTGGG	76	[32]
AXIN	Primer R Primer F	CCGTCGAAGTCTCACCTTTAATG GGTTTCCCCTTGGACCTCG	157	[28]
APC	Primer R Primer F	CTGAAGTTGAGCGTAATACCAGT AAAATGTCCCTCCGTTCTTATGG	222	[28]
CTNNB1	Primer R Primer F	CGAGTCATTGCATACTGTCCAT AAAGCGGCTGTTAGTCACTGG	215	[28]
SLCO2A1	Primer R Primer F	ACACTTCTGTGGTCACTCGTC GAGAGATTTGAATGTTGGACAAAGC	89	[24]

### Statistical analysis

Using a Wilcoxon signed-rank test, we compared the amounts of *B. fragilis* in the tumor and adjacent normal mucosa from paired samples. The Fisher exact test was used to assess the relationship between the ordinal categories of the number of bacteria and categorical data, such as age, sex, family history and disease stage. SPSS v.20.0 software (SPSS Inc., Chicago, IL, USA) and GraphPad Prism v.8.3.0 were used for the statistical analyses. In this study, statistical significance was defined as a two-tailed p-value < 0.05.

## Results

### Clinicopathological characteristics

The clinicopathological and demographic characteristics of 30 patients are shown in Table 2. The mean age of the patients was 57 (SD ± 13.87, range 26 to 78), and there was an equal number of men and women. The majority of patients were grade II (76.7%), while 13.3%, 6.7%, and 3.3% of cases were grades I, III, and IV, respectively. Overall, 27 patients (90%) were diagnosed with adenocarcinoma, and three patients (10%) had mucinous (colloid) adenocarcinoma. Notably, 66.7% of patients had colon cancer, whereas 33.3% had rectal cancer. Finally, only 13.3% of patients were social drinkers, and 10% were smokers.

**Table 2:** General and clinicopathological characteristics of Iranian patients with colorectal cancer (N = 30)

**General characteristics**		**Smoking status**	
Male/Female (n (%)) Age (Mean ± SD) Prior cancer Tumor size (Mean ± SD) Age of death (Mean ± SD) Weight loss (Mean ± SD) Height (Mean ± SD) Weight (Mean ± SD) Family history	15 (50%)/15 (50%) 56.40 ± 13.87, ranging from 26 to 78 3.3% (one patient with breast cancer) 5.9 ± 2.1, ranging from 2.5 to 11 58.75 ± 18.03, ranging from 33 to 79 8.66 ± 4.62, ranging from 2 to 16 166.5 ± 9.4, ranging from 153 to 183 66 ± 12.57, ranging from 39 to 95 40%	Non-smoker DX-smoker at diagnosis but discontinued Smoker Ex-smoker	80% 6.7% 10% 3.3%
		**Alcohol status**	
		Non-drinker Social drinker	86.7% 13.3%
**Site of primary**		**Invasion, Nodal status, and Tumor deposit**	
Cecum Ascending colon Transverse Colon Splenic Flexure Descending Colon Sigmoid Colon Rectosigmoid Rectum Colon, NOS	20% 10% 3.3% 3.3% 3.3% 13.3% 10% 33.3% 3.3%	Lymphatic Vascular Perineural Perineal Extramural Blood Vessel Extra-Nodal Extension Perforation Peritoneal Seeding	56.7% 53.3% 33.3% 3.3% 0 10% 10% 10%
**Grade**		**TNM staging**	
I: (Well Differentiated) II: Moderately Differentiated III: Poorly Differentiated IV: Undifferentiated	13.3% 76.7% 6.7% 3.3%	Stage I Stage IIA Stage IIB Stage IIIB Stage IIIC	13% 40% 6.7% 26.7% 13.3%
**Histology**			
Adenocarcinoma Mucinous (colloid) adenocarcinoma	90% 10%		
**Pathological T**		**Pathological N and Clinical Metastasis**	
T2 T3 T4	13.3% 76.7% 10%	N0 N1 N2 M0	60% 26.7% 13.3% 100%

### ***B. fragilis *quantification**

**Fig. 1:**
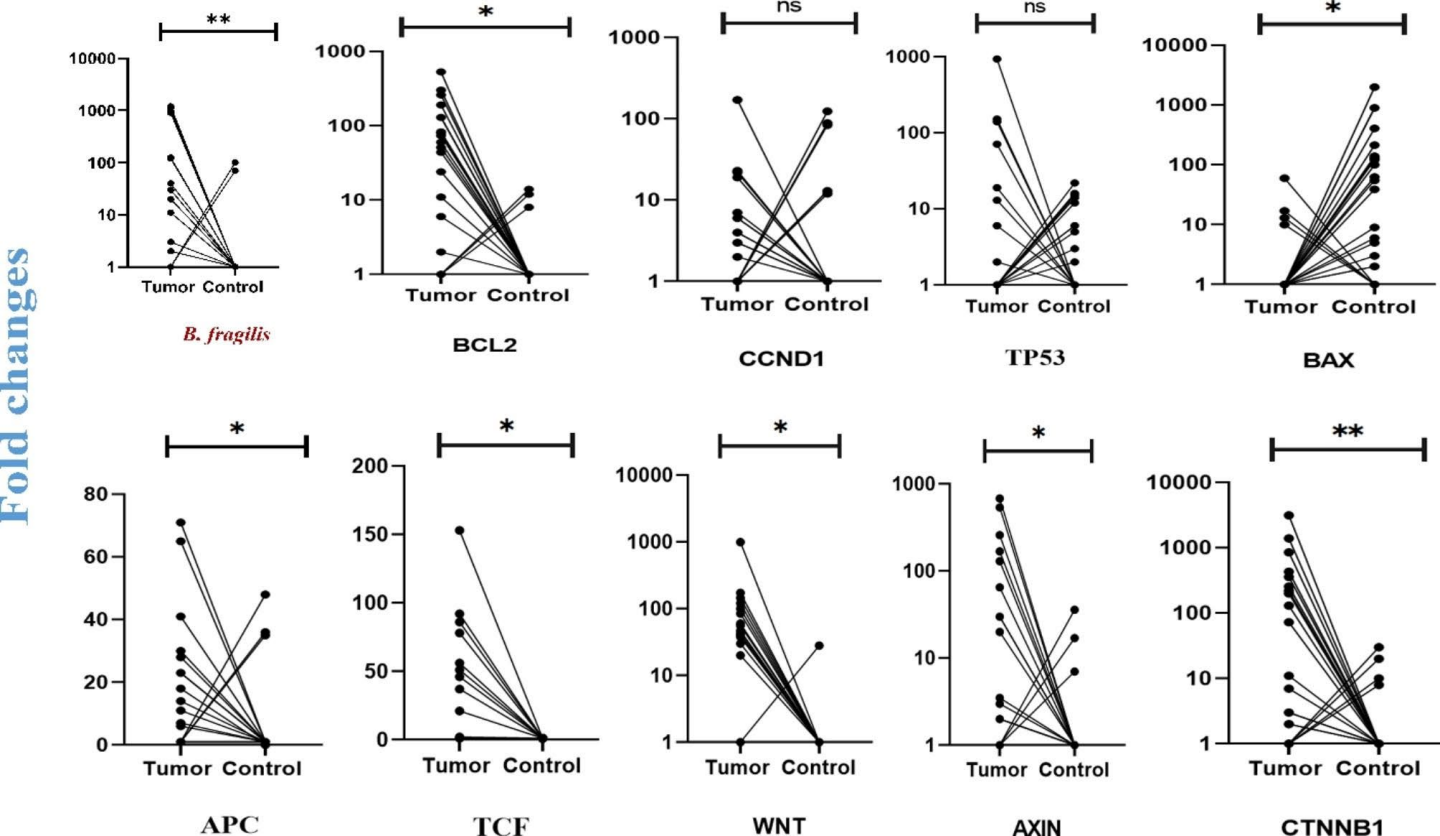
Relative quantification of *B. fragilis* and signaling pathway genes. The relative quantity of *B. fragilis* (n = 30, p < 0.01**) was significantly higher in CRC samples than in adjacent normal tissues. The relative quantity of CTNNB1 (n = 30, p < 0.01**), BCL2, APC, TCF, WNT, and AXIN (n = 30, p < 0.05*) was significantly higher in CRC samples than in the adjacent normal tissues. BAX was higher in control tissues than in tumor tissues (p < 0.05*). On the other hand, there was no significant difference (ns) in the relative quantification of TP53 and CCND1 (n = 30, p > 0.05^ns^) between CRC samples and non-CRC tissues

In this study, the relative quantification of *B. fragilis* in tumor tissues compared to the adjacent healthy tissue was determined by qPCR. The median abundance of *B. fragilis*, as evaluated by 2^−ΔΔCT^ (p < 0.01), was significantly higher in tumor tissues than in adjacent normal tissues (Fig. 1). *B. fragilis* was detected in 100% and 86% of the tumor and adjacent healthy samples, respectively. Overall, in 19 tumor samples, *B. fragilis* was higher than in adjacent healthy tissues. Noteworthy, we categorized CRC cases with detectable *B. fragilis* as low or high based on the median cut-point amount of this bacterium [25].

**Fig. 2:**
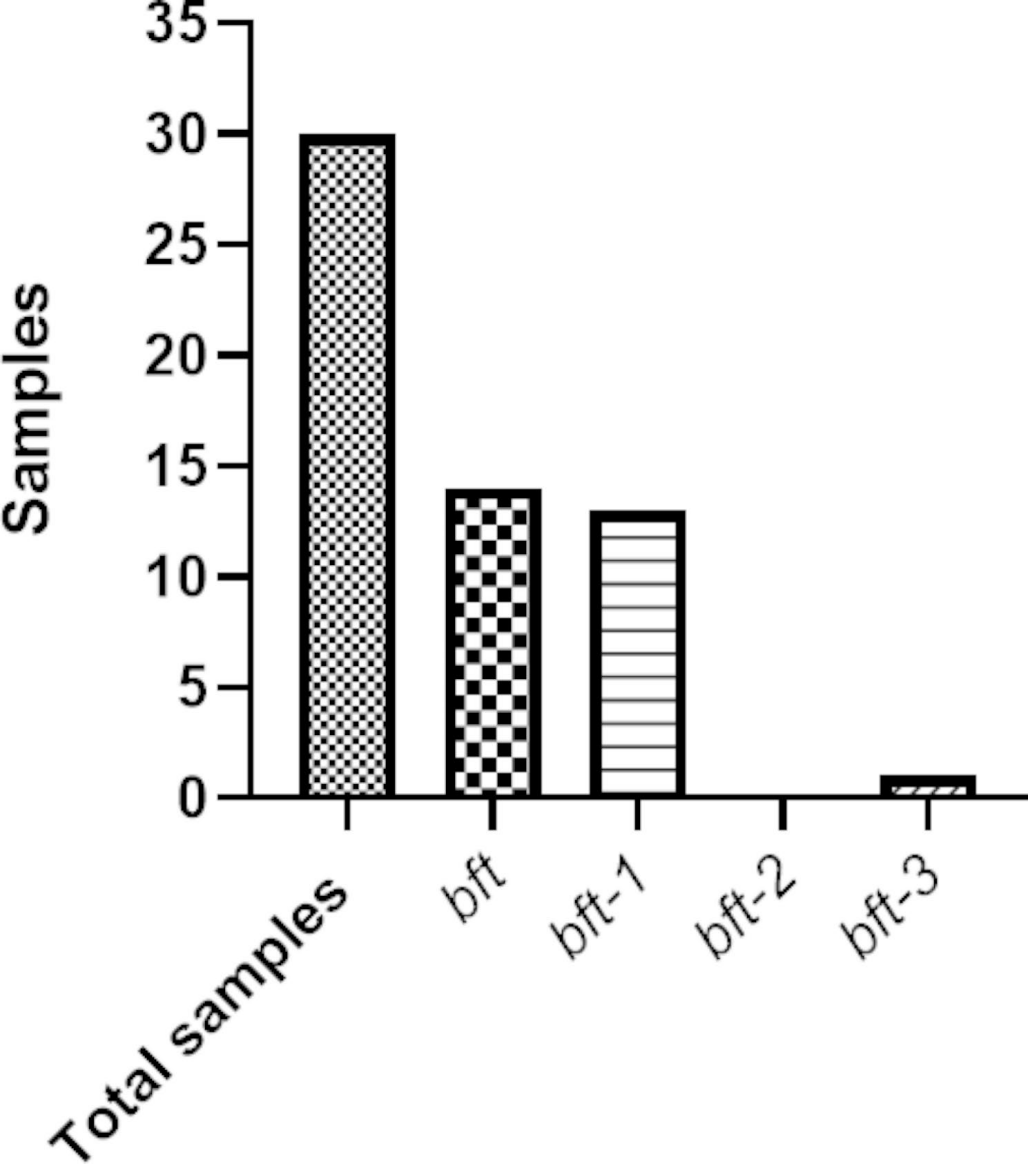
Prevalence of *B. fragilis* toxin isotypes among Iranian patients with CRC. *bft-1* was the most prevalent toxin in the samples (92.8%), one sample had *bft-3* (7.2%), and no sample harbored *bft-2* toxin

According to the PCR results, bft toxin was detected in 14 (47%) samples, and the most prevalent toxin isotypes in the samples were *bft-1* (92.9%), and one sample had *bft-3* (7.1%) and no sample harbored *bft-2* toxin (Fig. 2).

### WNT/ β-catenin, TP53 and BCL2 pathway gene expression

In this study, the expression level of the genes APC, TCF, WNT, AXIN, CTNNB1, BCL2, BAX, CCND1, and TP53 was investigated through qPCR and 2^−ΔΔCT^. The results showed that BCL2, APC, TCF, WNT, AXIN, and CTNNB1 (p < 0.05) were significantly higher expressed in tumor tissues compared to adjacent healthy tissues, and BAX was higher in control tissues than tumor tissues (p < 0.05). There is no significant difference between tumoral and normal samples for TP53 and CCND1 (p > 0.05) (Fig. 1).

### **Association between *B. fragilis *and signaling pathway genes and clinicopathological characteristics**

The analysis showed that there is a significant correlation between the greater amount of *B. fragilis* and high levels of AXIN, CTNNB1, and BCL2 genes (p < 0.05). On the other hand, no significant correlation between *B. fragilis* and the clinicopathological characteristics of patients was observed (p > 0.05) (Table 3).

**Table 3:** Association of *B. fragilis* with signaling pathway genes and clinicopathological characteristics

Characteristics (numbers (%))				Correlation with *B. fragilis* (P value)
Clinicopathological	Age	< 50	6 (20%)	> 0.05
		> 50	24 (80%)	> 0.05
	Sex	Male	15 (50)	> 0.05
		Female	15 (50)	
	Stage	I	4 (13.3%)	> 0.05
		II	23 (76.7%)	
		III	2 (6.7%)	
		IV	1 (3.3%)	
	Histology	Adenocarcinoma	27 (90%)	> 0.05
		Mucinous	3 (10%)	
	Site of primary	Colon	20 (66.7%)	> 0.05
		Rectal	10 (33.3%)	
	Drinkers	4 (13.3%)		> 0.05
	Smokers	3 (10%)		> 0.05
Signaling genes	WNT/ *β*-catenin signaling pathway	WNT		> 0.05
		TCF		> 0.05
		APC		> 0.05
		CTNNB1		**< 0.05**
		AXIN		**< 0.05**
	Apoptotic Pathways	CCND1		> 0.05
		BCL2		**< 0.05**
		BAX		> 0.05
		TP53		> 0.05

## Discussion

CRC is one of the most prevalent malignancies in the world. Among the various risk factors for it, the microbiome’s role in its onset and development has lately received a lot of attention [23]. Studies have reported that bacteria such as *Streptococcus bovis*, *F. nucleatum, E. coli*, and *B. fragilis* are associated with CRC [3]. *B. fragilis* is a commensal anaerobic bacterium in the intestine that, under certain conditions, can become an opportunistic pathogen and cause a variety of diseases, including peritonitis, toxin-associated diarrhea, soft tissue infections, and pelvic, lung, and brain abscesses [33, 34].

In this study, the relative amount of *B. fragilis* in tumor tissues was determined compared to the adjacent healthy tissues. In addition, the correlation between this bacterium and the expression of cellular signaling pathway genes involved in CRC was investigated. Based on our research, this is the first study to evaluate the correlation between *B. fragilis* and the expression of cellular signaling pathways and genes involved in CRC. According to our findings, the relative amount of *B. fragilis* was significantly higher in tumor tissues than in adjacent healthy tissues.

Our finding is in agreement with another report in Iran on stool samples, which reports that *B. fragilis* was significantly higher in CRC samples compared to the control group [18]. *Zamani* et al. conducted a study on mucosal biopsy samples and detected *B. fragilis* in 63% of mucosal biopsy samples from patients and 81% of samples from healthy controls [35]. Another study that was conducted in Iran also identified *B. fragilis* in 66% and 60% of the tumor and healthy adjacent tissues, respectively. Statistical analysis showed a significantly higher amount of bacteria in cancerous tissues in comparison to the normal samples [24]. Therefore, according to the mentioned reports, the prevalence of *B. fragilis* is higher in the CRC sample in comparison to the healthy tissues. On the other hand, it is possible that bacterial dysbiosis might be linked to CRC carcinogenesis or that the increase of *B. fragilis* may have happened as a result of cancer [24]. Enterotoxin is an important virulence factor in *B. fragilis*. When ETBF is chronically colonized in the intestine, it can cause inflammation by stimulating the production of some cytokines. It also changes several signaling pathways in the intestine and causes DNA damage through the production of ROS, all of which play a role in CRC tumorigenesis [35–37].

There are three isotypes of bft: *bft-1*, *bft-2*, and *bft-3.* Based on different research carried out in various regions, *bft-1* was the most prevalent isotype in Iran, Turkey, and the USA [38]. Our results also indicated *bft-1* as the most abundant isotype in CRC samples. Furthermore, there was only one *bft-3*, while we did not detect *bft-2* in our samples. *bft-1* and *bft-2* were the two most prevalent isotypes, according to the findings of a recently published study [35]. This supports the finding by *Toprak* et al., who reported that the *bft-1* isotype was significantly higher in stool samples of patients with CRC, followed by *bft-2* [39]. However, there have been reports that *bft-2* is the most common isotype in stool or mucosal samples from CRC patients. Of course, it should be noted that according to studies, the *bft*-*2* isotype is more carcinogenic than *bft-1 in vitro* and in vivo [16, 18]. Collectively, the reasons why *bft-2* was not detected in our study could be different geographical regions, genetic backgrounds, or dietary habits [39].

Several studies have determined the association of ETBF with CRC [40]. To this end, *Rezasoltani* et al. detected a higher amount of ETBF in patients with tubular adenoma, in particular villous/tubulovillous polyps, compared with normal samples [41]. Our recent study also demonstrated that 15% of the *B. fragilis*-positive subjects had ETBF infections in both the tumoral and adjacent normal tissues [24]. Although studies have shown that ETBF is associated with human CRC, more studies should be done, and the interactions of other bacteria with this bacterium as well as the impact of other risk factors along with the toxin and the concentration of toxin produced in patients should be comprehensively investigated [40, 42].

In this study, we examined the association between *B. fragilis* and the expression of cellular signaling pathway genes. Currently, there are not many studies on the correlation between *B. fragilis* and changes in the expression of signaling genes in CRC. However, previous studies have revealed a connection between CRC and the WNT/β-catenin signaling pathway. Overactivation of the WNT/β-catenin pathway was reported in many cancers, including CRC [43–45]. Here, our findings showed APC, TCF, WNT, AXIN, and CTNNB1 genes were overexpressed in tumor tissues in comparison to the adjacent normal tissues. In the previous study, TCF expression was shown to be 83% lower in tumor tissue and adjacent mucosa as compared to normal mucosa. Increased expression of this gene has been observed in cancers such as hepatocellular, renal, and mammary gland cancers. The reason for these opposite results may be the heterogeneity of the studied population or the availability of different transcripts for the TCF gene [46]. In a study with 214 CRC tumor tissues, TCF was expressed in 99 (46%) of the samples. According to their analysis, this gene is considered a negative prognostic factor that has been associated with a low survival rate [47]. However, according to *Moghadamnia* et al., there was no significant difference in APC expression levels in tumor tissues compared to normal samples [48]. Additionally, APC is the gene in which the highest rate of mutation occurs, but still, due to the many mutations that occur both in CRC and in this gene, it cannot be considered a prognostic factor for CRC [23].

Inconsistent with our result, a previous study detected overexpression of WNT and CTNNB1 in CRC tissues compared with para-carcinoma tissues [49]. Notably, our findings revealed a significant relationship between a high amount of *B. fragilis* and high levels of AXIN and CTNNB1 expressions in the tumor tissues. A recently published study reported that virulence factors from different pathogenic bacteria, such as *Shigella species*, *Helicobacter pylori*, and *Salmonella enterica* serovar *Typhimurium*, apply a range of molecular strategies to modify the appropriate functioning of the WNT/β-catenin pathway. The results of a study showed that bft could increase MYC expression and TCF reporter activity, thereby enhancing the β-catenin stability. Actually, the authors proposed that bft protease activity on the extracellular domain of E-cadherin disrupts epithelial cell-to-cell contact. The result is that bft leads to the dissociation of E-cadherin from β-catenin, once released, β-catenin translocates to the nucleus where it forms a complex with TCF4, leading to MYC expression and cellular proliferation in the APC mutant cell lines HT29/C1 and SW48. Altogether, it seems bft could be associated with the dysregulation of the WNT/β-catenin pathway, and this pathway must be tightly regulated due to its important physiological role, as its dysregulation, which is brought on by *B. fragilis*, may alter cell proliferation, apoptosis, and inflammation-related CRC [20, 50, 51]. However, more confirmatory studies are needed in this field.

BAX protein, which is a member of the BCL2 protein family, is activated by TP53 to induce apoptosis [52]. BCL2 is an anti-apoptotic member and its expression is reduced in CRC [53]. In the present study, the expression of BCL2 was higher in CRC tissues than in normal samples. In contrast with our result, *Gil* et al. reported that BCL2 and BAX expressed lower and higher in cancerous tissues than in normal adjacent tissue samples, respectively [54]. In another study, the authors reported that the expression of BAX in cancer tissues is significantly higher than in healthy tissues [55]. Also, statistical analyzes in this study showed that there is a significant relationship between high levels of *B. fragilis* and high levels of BCL2 expression in tumor samples. In this regard, previous studies reported that the use of different probiotics, such as *Lactobacillus plantarum* and *Lactobacillus acidophilus* could decrease the expression of anti-apoptotic BCL2 in CRC cell lines [56, 57]. Therefore, it seems pathogenic bacteria, such as ETBF, can enhance the expression of the BCL2 and the chance of tumorigenesis in CRC. Unfortunately, recent studies did not evaluate the interaction of CRC-associated bacteria with anti-apoptotic genes; therefore, this possible mechanism of carcinogenesis should be considered in future studies.

Furthermore, TP53 is another pathway involved in the CRC process. This gene is a transcription factor that converts stress signals into cellular actions such as cell cycle arrest, DNA repair, and apoptosis [58]. Under physiological conditions, TP53 is expressed at a low level, but its expression level increases under cellular stress conditions. During CRC carcinogenesis, TP53 mutations play a vital role in the adenoma-carcinoma progression [59]. In our study, we found no significant alteration of TP53 between tumor tissues and adjacent healthy tissues. On the other hand, previous studies have shown that TP53 is significantly increased in CRC samples [60, 61]. Noteworthy, it was reported that mutations in the TP53 gene cause the accumulation of P53 protein [62]. Different results from other studies have been obtained due to the difference in the study population, the type of method used, the difference in the determined cut-off, and also the stage of the disease [61]. It should be noted that changes in TP53 expression occur more often in younger patients, less than 40 years old, than in older patients [58]. Another of our results is related to CCND1, in which statistical analysis did not show any significant alteration of this gene between tumor and healthy tissue. In contrast previous studies have identified increased expression of CCND1 in tumor tissues [63, 64]. *Albasri* et al. reported that overexpression of CCND1 is related to advanced stages of tumor and poor survival rate of CRC [63]. Studies have shown that the overall amplification of CCND1 is related to lymph node metastasis and invasive tumor histology [63, 65]. The results of our study regarding this gene could be due to the fact that our samples are grade II; however, this issue should be evaluated exactly in future studies.

## Conclusion

The results showed that *B. fragilis* levels increased significantly in the tumor samples compared to the adjacent healthy tissues. Additionally, the analysis showed that there is a significant relationship between a greater amount of this bacterium and the high-level expression of AXIN, BCL2, and CTNNB1 genes in CRC samples. Maintaining tight control over WNT/β-catenin signaling is crucial, since the dysregulation of this pathway due to various stimuli, such as *B. fragilis*, can result in changes in cell proliferation, apoptosis, and inflammation-associated malignancy. To this end, more research in this field should be done on a larger population to investigate the exact interaction between *B. fragilis* and its toxin and WNT/β-catenin signaling pathways involved in CRC. Once these interactions are identified, the development of targeted medications to neutralize the virulence factors would be the next logical step.

## Data Availability

Data sharing does not apply to this article as no datasets were generated during the current study.
